# Reduced Dose of Post-Transplant Cyclophosphamide with Tacrolimus for the Prevention of Graft-versus-Host Disease in HLA-Matched Donor Peripheral Blood Stem Cell Transplants: A Prospective Pilot Study

**DOI:** 10.3390/cancers16142567

**Published:** 2024-07-17

**Authors:** Alex Juárez, María Queralt Salas, Alexandra Pedraza, María Suárez-Lledó, Luís Gerardo Rodríguez-Lobato, María Teresa Solano, Anna Serrahima, Meritxell Nomdedeu, Joan Cid, Miquel Lozano, Paola Charry, Jordi Arcarons, Noemí Llobet, Laura Rosiñol, Francesc Fernández-Avilés, Montserrat Rovira, Carmen Martínez

**Affiliations:** 1 Hematopoietic Stem Cell Transplantation Unit, Hematology Department, Institute of Cancer and Blood Disease (ICAMS), Hospital Clínic de Barcelona, 08036 Barcelona, Spain; alexjuarezc@gmail.com (A.J.); mqsalas@clinic.cat (M.Q.S.); msuarezl@clinic.cat (M.S.-L.); lgrodriguez@clinic.cat (L.G.R.-L.); mtsolano@clinic.cat (M.T.S.); serrahima@clinic.cat (A.S.); jarcarons@recerca.clinic.cat (J.A.); nllobet@clinic.cat (N.L.); lrosinol@clinic.cat (L.R.); ffernandez@clinic.cat (F.F.-A.); mrovira@clinic.cat (M.R.); 2 Hemotherapy and Hemostasis Department, Institute of Cancer and Blood Disease (ICAMS), Hospital Clínic de Barcelona, 08036 Barcelona, Spain; acpedraza@clinic.cat (A.P.); mnomdedeu@clinic.cat (M.N.); 3 August Pi i Sunyer Biomedical Research Institute—IDIBAPS, 08036 Barcelona, Spain; jcid@clinic.cat (J.C.); mlozano@clinic.cat (M.L.); charry@clinic.cat (P.C.); 4 Apheresis and Cellular Therapy Unit, Hemotherapy and Hemostasis Department, Institute of Cancer and Blood Disease (ICAMS), Hospital Clínic de Barcelona, 08036 Barcelona, Spain; 5 Institute Josep Carreras, Hospital Clínic de Barcelona, 08036 Barcelona, Spain

**Keywords:** allogeneic hematopoietic stem cell transplantation, post-transplant cyclophosphamide, graft-versus-host disease, GVHD, reduced-dose PTCY

## Abstract

**Simple Summary:**

High-dose post-transplant cyclophosphamide is effective in preventing graft-versus-host disease (GVHD) but is associated with adverse outcomes such as delayed engraftment, infections, and cardiac issues. This pilot study evaluated the efficacy and toxicity of reduced-dose PTCY (40 mg/kg/day) in patients undergoing HLA-matched allogeneic hematopoietic stem cell transplantation (alloHSCT). Neutrophil and platelet engraftment occurred at medians of 15 and 16 days, respectively. At day 100, the incidences of grade II–IV and III–IV acute GVHD were 18.2% and 4.5%, respectively, with no cases of grade IV acute GVHD or steroid-refractory disease. One-year incidence of moderate-severe chronic GVHD was 6.4%. Both incidences, acute GVHD and chronic GVHD, are similar to our previous experience with higher doses of PTCY. Two-year overall survival and relapse-free survival were 77.1% and 58.3%. There were low incidences of infections and only one early cardiac event. These results suggest that reduced-dose PTCY provides adequate immunosuppression with a low toxicity profile.

**Abstract:**

PTCY 50 mg/kg/day on days +3/+4 is an excellent strategy to prevent GVHD. However, its use is associated with adverse outcomes such as delayed engraftment, increased risk of infection, and cardiac complications. This pilot study evaluates the efficacy and toxicity of a reduced dose of PTCY (40 mg/kg/day) combined with tacrolimus in 22 peripheral blood HLA-matched alloHSCT patients. At day +100, the cumulative incidences of grade II–IV and III–IV acute GVHD were 18.2% and 4.5%, respectively. No grade IV acute GVHD or steroid-refractory disease was observed. The cumulative incidences of all-grade and moderate-severe chronic GVHD at 1-year were 11.4% and 6.4%, respectively. No patient died from transplant-related complications. Two-year OS and RFS were 77.1% and 58.3%, respectively. All patients engrafted, with neutrophil and platelet recovery occurring at a median of 15 (IQR 14–16) and 16 days (IQR 12–23), respectively. The cumulative incidences of bloodstream bacterial infections, polyomavirus BK hemorrhagic cystitis, HHV6 reactivation, CMV reactivation, and fungal infections were 13.6%, 9.1%, 9.1%, 4.6%, and 6%, respectively. Only one early cardiac event was observed. These results suggest that PTCY 40 mg/kg/day on a +3/+4 schedule provides adequate immunosuppression to allow for engraftment and prevent clinically significant GVHD with a low toxicity profile.

## 1. Introduction

Post-transplant high-dose cyclophosphamide (PTCY) is an increasingly popular approach for graft-versus-host disease (GVHD) prophylaxis in allogeneic hematopoietic stem cell transplantation (alloHSCT). This is true not only in the context of haploidentical alloHSCT [1,2], but also in transplants from HLA-matched sibling donors (MSD) and unrelated donors (MUD) [3,4,5,6,7,8]. Several studies have shown that the use of PTCY at a dose of 50 mg/kg/day on days +3 and +4 (+3/+4) in combination with tacrolimus (Tac) and mycophenolate mofetil (MMF) significantly reduces the incidence of global and severe forms of acute and chronic GVHD [1,2,3,4,5,6,7,8]. Due to the positive results obtained with this combination, PTCY 50 mg/kg/day on days +3/+4 has become the standard dose for most centers.

PTCY combined with Tac and MMF was implemented for GVHD prevention at our institution for PB stem cells (PBSC) haplo-HSCT in 2013. In the subsequent years, this prophylaxis was progressively extended to alloHSCT performed from HLA-matched donors with notable success. Consecutively, secondary to the positive results obtained from this prophylaxis, and for the purpose of reducing side effects, the use of MMF on this setting was discontinued. Since then, PTCY plus Tac became our institutional GVHD prophylaxis for alloHSCT performed from MSD and MUD.

Similarly to what has been observed in other institutions where PTCY-based prophylaxis has been implemented, GVHD incidences have been appropriately prevented regardless selected donor type. However, the use of this prophylaxis has also been associated with non-desired outcomes such as delayed engraftment and immune reconstitution, increased risk of infections, and specific organ toxicities such as cardiac complications [6,7,8,9,10,11,12,13,14]. Recent studies have investigated whether a reduced dose of PTCY could provide comparable prevention of GVHD with a better toxicity profile. Although preliminary, the results from these studies, conducted mainly in adults undergoing haploidentical peripheral blood (PB) alloHSCT, have suggested that PTCY doses can be safely lowered in patients at risk for specific toxicities, such as older adults or those with a prior cardiac history, when combined with other immunosuppressive agents like anti-thymocyte globulin (ATG) and calcineurin inhibitors with and without MMF in some of these studies.

Based on the positive results observed in our program using PTCY combined with Tac for the prevention of GVHD in HLA-matched PBSC alloHSCT, we designed for this transplant setting an institutional protocol with reduced doses of PTCY at 40 mg/kg/day on days +3 and +4, with Tac as a complementary immunosuppressive agent in September 2021 with the aim of maintaining the efficacy of PTCY-based prophylaxis in preventing GVHD while reducing its toxicity profile. Concomitantly, the use of 300 mcg/24 h of granulocyte colony-stimulating factor (G-CSF) from day +7 until neutrophil recovery was implemented to shorten the aplastic phase and reduce early infectious complications. After performing 22 alloHSCT under this new institutional transplant protocol, we present the preliminary results obtained from the implementation of this protocol in adults undergoing HLA-matched alloHSCT.

## 2. Patients and Methods

### 2.1. Study Design

This is a single-center prospective study aimed at reducing PTCY toxicity and shortening the hematopoietic recovery period after transplantation without increasing the risk of GVHD. Two measures were implemented to achieve this goal: reducing the PTCY dose from the standard 50 mg/kg/day on days +3/+4 to 40 mg/kg/day on days +3/+4, and systematically adding 300 mcg/24 h of G-CSF to all patients starting on day +7 after transplantation.

This study included adult patients between the ages of 18 and 70 who had been diagnosed with hematologic malignancy and had undergone alloHSCT from a 10/10 HLA-identical related or unrelated donor. The eligibility criteria were as follows: Eastern Cooperative Oncology Group performance status of 2 or less, left ventricular ejection fraction (LVEF) of 35% or higher, forced expiratory volume in 1 s and forced vital capacity of at least 40% of predicted, and adequate hepatic function (total bilirubin of 3.0 mg/dL or less or no clinically significant liver disease). Patients with previous alloHSCT were excluded.

All donor/recipient pairs were typed by high resolution for allelic level for HLA-A, HLA-B, HLA-C, HLA-DRB1, and HLA-DQB1. Other donor selection criteria, in order of priority, were as follows: matched cytomegalovirus (CMV) IgG serologic status; a male donor for a male recipient; major ABO compatibility; and minor ABO compatibility. The protocol received institutional review board approval, and all participants provided signed informed consent.

### 2.2. Treatment Protocol and Supportive Care

Specific conditioning regimens used were based on the type of hematologic disease and patient characteristics in accordance with institutional protocols (Table 1). Patients aged >50 years or previously submitted to an autologous HSCT received a reduced-intensity conditioning (RIC) regimen; otherwise, myeloablative conditioning (MAC) regimens were administered.

All patients received unmanipulated PBSC grafts. The optimal CD34^+^ cells per kilogram of recipient weight was set between 4 and 8 × 10^6^/kg, and according to our institutional protocol, the maximum CD34^+^ cell dose was capped at 8 × 10^6^/kg. Remaining doses of stem cell products were cryopreserved.

GVHD prophylaxis consisted of PTCY at a dose of 40 mg/kg/day on days +3/+4, along with Mesna at 120% of the cyclophosphamide dose (divided into three doses), followed by Tac (0.03 mg/kg as a 24 h IV perfusion) from day +4. Serum levels of Tac were maintained at therapeutic levels (between 5 and 15 ng/mL) until day +90 and then tapered if aGVHD grade II–IV was absent. No adjustments of Tac doses were performed according to the Disease Risk Index (DRI). ATG or alemtuzumab were not administered.

G-CSF was administered from day +7 until 3 consecutive days with an absolute neutrophil count (ANC) of >1 × 10^9^/L after transplantation.

Standard antimicrobial prophylaxis consisted of levofloxacin from day 0 until neutrophil engraftment, fluconazole until day +60, and acyclovir until day +365 (for patients who were seropositive for herpes simplex virus or herpes zoster virus). Standard *Pneumocystis jirovecii* prophylaxis was used until CD4^+^ T cell recovery (>200 cells/μL) and/or until immunosuppression was discontinued. All patients with positive CMV serology received letermovir from day +7.

CMV quantitative polymerase chain reaction (PCR) was performed weekly through to at least day +60, and pre-emptive therapy was initiated if viral reactivation was detected (a PCR result above 1000 UI/mL or two consecutive rising values), according to standard recommendations. As the patients did not receive ATG, measurement of Ebstein-Barr virus (EBV), herpes human virus 6 (HHV6) and BK-virus occurred according to clinical suspicion. Fungal infection monitoring was performed in patients’ plasma samples with galactomannan antigenemia weekly according to the frequency of patient visits, and until withdrawal of immunosuppression. Infectious treatment followed standardized guidelines.

### 2.3. Definitions

Neutrophil recovery was defined as the first of 3 consecutive days with an ANC of >0.5 × 10^9^/L after transplantation. Platelet recovery was defined as a platelet count of >20 × 10^9^/L without transfusion in the 7 preceding days. Donor chimerism was assessed by PCR-based amplification of polymorphic short tandem repeat regions in peripheral blood samples taken on days +30, +60, and +90. Chimerism analysis was performed using separated myeloid and T cell lymphoid fractions whenever possible. After a dilutional assay conducted in our laboratory, complete chimerism is considered when >95% donor cells are detected by flow cytometry [15].

Primary graft failure was defined as the absence of ANC recovery (>0.5 × 10^9^/L) at day +28, which was maintained for 3 consecutive days, with a platelet count <20 × 10^9^/L, hemoglobin level <80 g/L, and the need for transfusion support.

Toxicity was scored using Common Terminology Criteria for Adverse Events (CTCAE) version 5. Hepatic sinusoidal obstruction syndrome was graded using the Baltimore criteria, and hemorrhagic cystitis was considered significant when macroscopic hematuria was present in the absence of other clinical conditions. Acute GVHD was scored using the MAGIC criteria [16]. The hematopoietic cell transplantation-specific comorbidity index (HCTCI) was determined as described by Sorror et al. [17]. Disease risk of the patients was determined according to the refined DRI [18].

### 2.4. Statistical Analysis

Categorical variables related to patients, disease, and transplant procedure were represented as frequencies and proportions. Quantitative variables were summarized as median and interquartile range (IQR) or range. Non-relapse mortality (NRM) was defined as the time from alloHSCT to death in the absence of prior relapse/progression. The relapse rate was calculated as the time from alloHSCT to relapse/progression. NRM and relapse rate events were considered as competing risks. Relapse-free survival (RFS) was defined as the time from alloHSCT to relapse/progression or death from any cause, and overall survival (OS) was defined as the time from alloHSCT to death from any cause.

The cumulative incidence of GVHD was estimated considering death and relapse as competing events. OS, RFS, and GVHD-free relapse-free survival (GRFS) were calculated using the Kaplan–Meier product-limit method. GVHD and GRFS were calculated accounting for death, relapse, grade III–IV acute GVHD, and moderate/severe chronic GVHD, as events. NRM and cumulative incidence of relapse (CIR) and the cumulative incidence of GVHD were estimated using the cumulative incidence method. Relapse was considered a competing event in the analysis of NRM, and death without relapse was considered a competing event in the study of CIR.

Statistical significance was defined at the 0.05 level. Analysis was performed using the SPSS version 28.0 (SPSS INC, Chicago, Illinois, USA) and R software (R version 4.3.2).

## 3. Results

### 3.1. Characteristics of the Patients

The 22 patients with hematological malignancies who underwent an HLA-matched related or unrelated alloHSCT from September 2021 to May 2021 at our institution were considered eligible to receive the modified institutional transplant protocol composed by reduced doses of PTCY and tacrolimus and G-CSF from day +7 to neutrophil recovery and included in this study.

Table 1 describes baseline patients and alloHSCT procedure characteristics. The median age of the study population was 53 years (IQR 18–68). Acute leukemia (54%) and myeloproliferative neoplasms (19%) were the most frequent transplant indications. DRI could be calculated in 18 patients with 50% of them being classified into low- and intermediate-risk categories and 50% into high- and very-high-risk categories. PBSC was used in all cases as graft source. Fourteen patients (64%) received transplants from MUD.

### 3.2. Engraftment and Chimerism

Neutrophil engraftment was achieved in all patients with a median of 15 days (IQR 14–16 days) and a median use of GSF of 9 days (IQR 7–11 days). Platelet engraftment occurred at a median of 16 days (IQR 12–23 days), with 68.2%, 81.8%, and 95.5% of patients having a count of >50,000 platelets at +30, +60, and +90 days after alloHSCT, respectively. The medians of red cell and platelet transfusions administered during the first 30 days after the stem cell infusion were 2 units (IQR: 0–8) and 3 pools (IQR: 1–8), respectively.

No patient experienced primary graft failure. ≥95% donor chimerism in CD3^+^ T-lymphocytes and unsorted cells was achieved in 13/19 (68%) and 21/22 (95%) evaluable patients, respectively, by day +60.

### 3.3. Toxicity and Infectious Complications

The median hospitalization period following transplantation was 32 days (IQR 29–42 days).

Ninety-one percent of the patients presented with mucositis, with seven (32%) presenting with grade 2, two (9%) with grade 3 and three (14%) with grade 4 mucositis. Among the patients with grade 3 and 4 mucositis, two had received MAC and three RIC regimes.

The cumulative incidences of infectious complications within the first 100 days were 13.6% for bloodstream bacterial infections, 9.1% for BK-virus hemorrhagic cystitis (HC), 9.1% for HHV6 reactivation, 4.6% for CMV reactivation, and 4.6% for fungal infection. No reactivations of the Epstein–Barr virus (EBV) were observed, and there were no cases of post-transplant lymphoproliferative disorder (PTLD). Below, we describe the infectious events observed throughout the follow-up period after the transplant.

Five patients (23%) experienced bloodstream bacterial infections, including *Klebsiella pneumoniae* (n = 2), *Staphylococcus epidermidis* (n = 2), and Methicillin-Resistant *Staphylococcus aureus* (n = 1). All infections were resolved with antibiotics. Two patients had fungal infections, one with invasive pulmonary aspergillosis and the other with nasopharyngeal mucormycosis. Both infections were successfully treated with antifungal therapy. Surgery was also performed in the case of mucormicosis. CMV reactivation occurred in 5 out of 18 (23%) seropositive patients at a median of 46 days (range 10–132) after alloHSCT. One (4.6%) patient developed CMV disease with gastrointestinal involvement. All patients responded to antiviral therapy. Two patients (9%) presented HHV6 reactivation in gastrointestinal tissue. One patient required specific treatment with cidofovir and presented a successful response. Nine patients (43%) had viral upper respiratory infections, including SARS-CoV-2 (n = 4, 18%), rhinovirus (n = 3, 14%), and bocavirus (n = 1, 5%), which were treated symptomatically and self-limited.

Of the total patients, 23% developed HC, all of which were related to polyomavirus BK infection. Three patients had grade 1 HC, one had grade 2, and one had grade 4. All patients were first treated with hyperhydration. Additionally, the patient with grade 4 HC, who had a recent medical history of local radiotherapy for prostatic adenocarcinoma, was initiated on and required antiviral treatment with intravenous foscarnet and required urological surgical measures. The remaining patients were treated with hyperhydration alone.

The cumulative incidence of early cardiac toxicity at day +100 was 4.5% (95% CI, 0.3–19.4). Only one early cardiac event was reported: a patient with a history of severe cardiac ischemic disease developed left ventricular systolic dysfunction on day +64 post-transplant. Specific treatment was initiated by the Cardiology Department with a progressive improvement of the left ventricular ejection fraction during the subsequent months.

### 3.4. Incidence of Acute GVHD and Chronic GVHD

**Figure 1:**
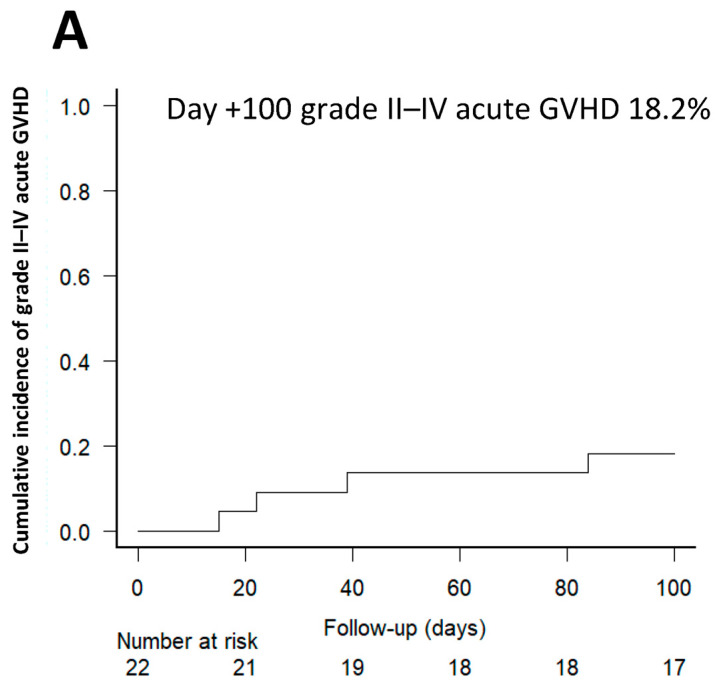

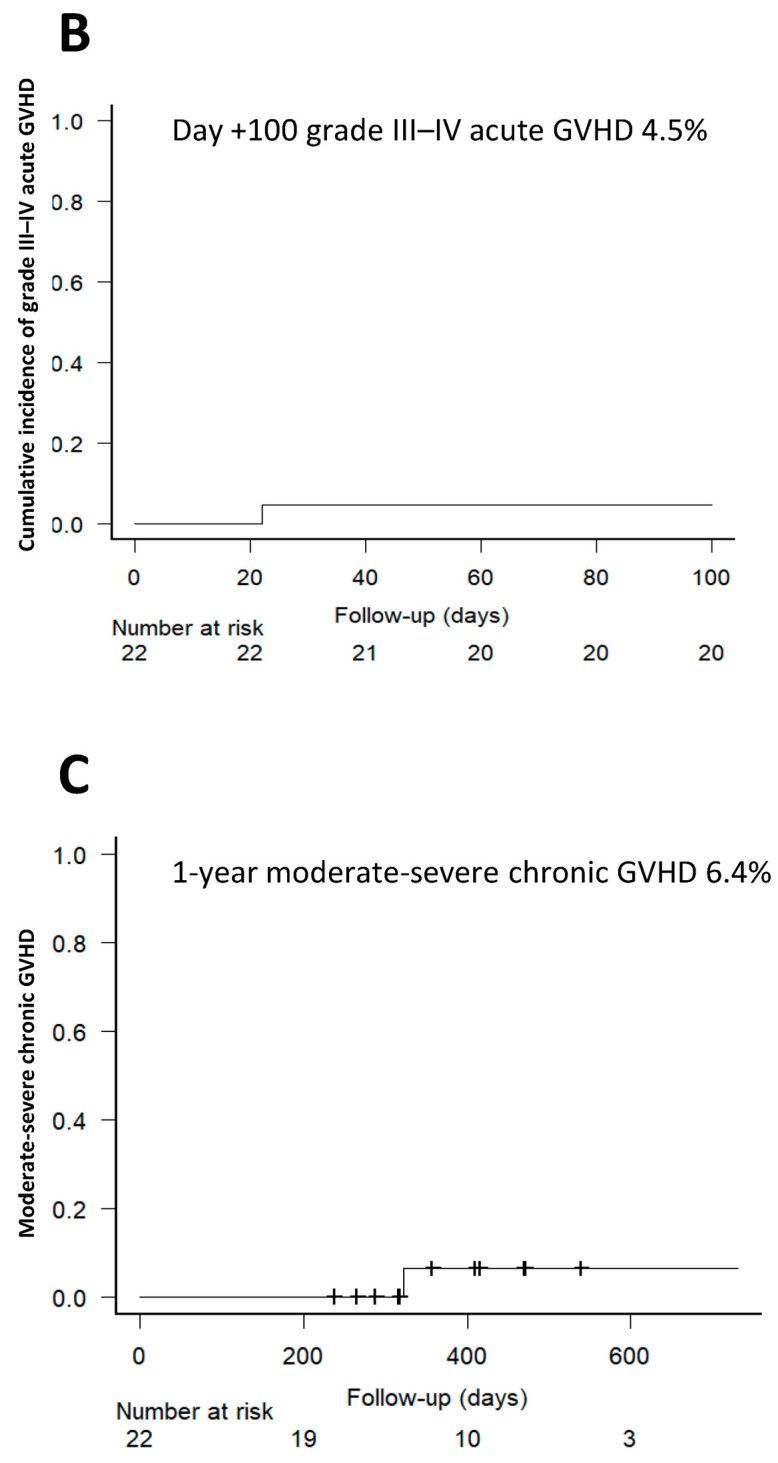
Cumulative incidence of grade II–IV (**A**) and III–IV (**B**) acute GVHD, and moderate-severe chronic GVHD (**C**) after alloHSCT.

At day +100, the cumulative incidences of grades II–IV and III–IV acute GVHD were 18.2% (95% CI, 5.5–36.8) and 4.5% (95% CI, 0.3–19.4), respectively (Figure 1A,B). Two patients presented acute GVHD intestinal involvement. GVHD were treated following standard guidelines. In patients with aGVHD, tacrolimus was maintained at therapeutic levels in all cases and treatment with 2 mg/kg/day of corticosteroids were initiated in all patients with grades II–IV aGVHD. No grade IV acute GVHD or steroid-refractory disease was observed. The cumulative incidences of all-grade and moderate-severe chronic GVHD at 1 year were 11.4% (95% CI, 17.0–31.6) and 6.4% (95% CI, 0.4–26.2) (Figure 1C). One patient was presented with moderate/severe cGVHD requiring first-line treatment with corticosteroids and calcineurin inhibitors with stabilization of the cGVHD.

### 3.5. Disease Relapse and Post-Transplant Outcomes

The median follow-up after transplant was 14 months (range 6–28). Seven patients experienced a relapse during the follow-up period after alloHSCT at a median of 224 days (range 56–525) after transplantation. The patients who relapsed had pre-transplant diagnoses of AML (partial remission, high-risk DRI), chronic eosinophilic leukemia (first complete remission, unclassifiable DRI), mycosis fungoides (partial remission, unclassifiable DRI), Richter’s syndrome (partial remission, intermediate-risk DRI), myelomonocytic leukemia (partial remission, high-risk DRI), AML (third complete remission, high-risk DRI), and myelodysplastic syndrome (partial remission, high-risk DRI). The estimated relapse rate at 1 and 2-year were 23.4% and 31.8%, respectively. No patient died from transplant-related complications.

**Figure 2:**
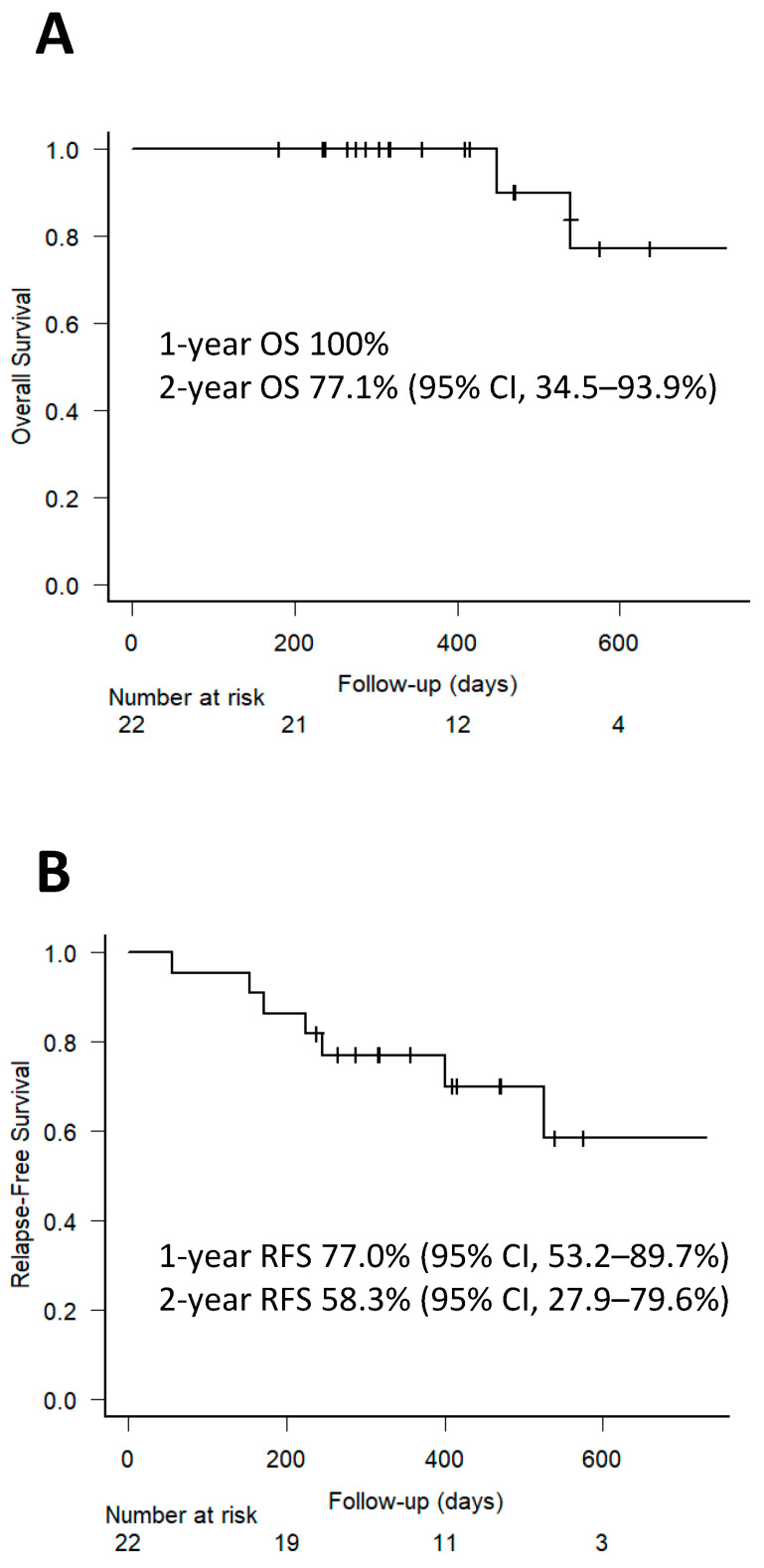
Estimated probabilities of overall survival (**A**) and relapse-free survival (**B**).

The estimated probabilities of OS at 1 and 2-year were 100% and 77.1% (95% CI, 34.5–93.9%), respectively (Figure 2A). The estimated probabilities of RFS at 1 and 2-year were 77.0% (95% CI, 53.2–89.7%) and 58.3% (95% CI, 27.9–79.6%), respectively (Figure 2B).

## 4. Discussion

In this pilot study, the safety and efficacy of PTCY at a reduced dose of 40 mg/kg/day on days +3/+4 combined with standard doses of Tac were assessed in recipients of MRD and MUD PBSC alloHSCT. The results revealed low incidences of grade II–IV and grade III–IV acute GVHD, and moderate-severe chronic GVHD (18.2%, 4.5%, and 6.4%, respectively), comparable to the standard PTCY dose of 50 mg/kg/day. No cases of grade IV acute GVHD or steroid-refractory disease were observed. Furthermore, none of the patients presented primary graft failure, indicating that PTCY at 40 mg/kg/day combined with Tac is both effective and safe.

In current clinical practice, the standard dose of PTCY at 50 mg/kg/day and its timing have been derived partly from murine MHC-matched skin allografting models and partly from empirical approaches. Recently, Wachsmuth et al. investigated the impact of varying doses of PTCY (5, 10, 25, 50 mg/kg/day) and timing (post-transplant days from +1 to +8) on its effectiveness in mitigating GVHD severity in a murine MHC-haploidentical alloHSCT model [19,20]. Overall, their findings suggest that PTCY reaches its maximal efficacy when given on day +4, and PTCY given on day +4 alone may be as effective as varied doses on days +3/+4. Notably, a PTCY dose of 25 mg/kg/day on day +4 was found to be equivalent to 25 mg/kg/day administered on days +3/+4 in preventing severe GVHD. These data support the initiation of clinical trials aimed at exploring PTCY dose de-escalation.

In haploidentical transplantation, several studies have evaluated reduced doses of PTCY, either alone or in combination with ATG. This setting carries a higher risk of GVHD and graft failure compared to MRD or MUD transplant recipients. PTCY doses vary from 40 mg/kg/day on days +3/+4 to 14.5 mg/kg/day on the same days, usually in combination with two to four immunosuppressant drugs [21,22,23,24]. Overall, the results of these studies show that PTCY dose de-escalation is feasible, allows hematopoietic engraftment and does not increase the risk of GVHD compared to standard doses.

Limited information, summarized in Table 2, is available on the use of reduced doses of PTCY with or without ATG in the setting of HLA-mismatched, MRD, and MUD alloHSCT.

In the protocol by Sun et al., the GVHD prophylaxis regimen consisted of a single dose of PTCY 50 mg/kg on day +3, ATG, CsA and MMF [25]. The incidences of grade II–IV acute GVHD at +100 days and mild-to-moderate chronic GVHD within 1 year were 6.2% and 11.5%, respectively. The results observed in our study using a simpler GVHD prophylaxis regimen, with PTCY 40 mg/kg/day on days +3/+4 and Tac instead of four immunosuppressants, were quite similar (4.5% for grade II–IV acute GVHD and 11.4% for all-grade chronic GVHD). In the second study, a comparison was made between PTCY 20 mg/kg on days +3/+4 with low-dose ATG and a quadruplet ATG-based regimen using ATG, methotrexate, CsA, and MMF in patients undergoing HLA-identical MUD-PBSC transplants [26]. The incidence rates of grade II–IV acute GVHD and chronic GVHD at 2 years were significantly reduced in the low-dose PTCY-ATG cohort (24.5% vs. 47.1%; 14.1% vs. 33.3%), indicating a positive effect of this treatment approach. According to these and other studies, the combination of low-dose PTCY with low-dose ATG could be a valid approach for GVHD prevention. However, many questions remain about potential toxicities, immune recovery, and infectious risks, specifically, for EBV reactivation and EBV-PTLD, which have been reported to be higher with ATG.

Three studies evaluated reduced doses of PTCY without the addition of ATG in recipients of MRD or MUD transplants [26,27,28,29]. Solterman et al. conducted a retrospective analysis of PTCY 40 mg/kg/day on days +3/+4, followed by CsA and MMF, in 22 patients who underwent 1-antigen HLA-mismatched unrelated donor transplant [27]. The authors compared this group with 58 patients who received ATG, CsA, and either methotrexate or MMF. The PTCY group had a significantly lower incidence of acute GVHD grade II–IV (15% vs. 50%), while chronic GVHD was similar (26% vs. 35%). In our study using Tac alone combined with PTCY 40 mg/kg/day, the incidence of acute GVHD grade II–IV was very similar (18.2%). Zhang et al. recently presented the results of a prospective phase I/II study using reduced doses of PTCY in transplant recipients from HLA-identical donors [28]. Following a 3 + 3 dose escalation design, they analyzed the results of PCTY 50 mg/kg/day on days +3/+4, 50 mg/kg on day +3 and 25 mg/kg on day +4, or 25 mg/kg on days +3/+4. Overall, none of the 29 patients enrolled in this study developed grade III–IV acute GVHD at 100 days. In patients who received PCTY at a dose of 25 mg/kg/day on days +3/+4, the incidence of grade II–IV acute GVHD at day +100 was 28.6%. Although this incidence was similar among the three groups of PCTY doses, it appears to be somewhat higher than that observed in our study, suggesting that the optimal dose of PCTY in combination with a single immunosuppressant (CsA or Tac) may be between 25 mg/kg/day and 40 mg/kg/day on days +3/+4.

Overall, the results of all these studies, together with our findings, suggest that the intensity of immunosuppression could be reduced, both the dose of PCTY and the number of immunosuppressive drugs associated with it, when PBSC grafts from HLA-matched donors are infused for alloHCT. Nevertheless, further studies are needed to determine PTCY appropriate dose and combination.

PTCY is associated with adverse effects including engraftment and immune reconstitution delays, increased risk of infection, and cardiac complications [6,7,8,9,10,11,12,13,14], suggesting the need for dose adjustment to reduce toxicity while maintaining efficacy in preventing GHVD. In a recent study, we showed that PTCY 50 mg/kg/day on days +3/+4 was associated with delayed neutrophil and platelet engraftment compared to non-PTCY regimens (20 vs. 16 days and 19 vs. 12 days, respectively) [30]. In the present study, neutrophil and platelet engraftment occurred more rapidly (median time of 15 and 16 days, respectively) than that previously reported with PTCY 50 mg/kg/day [30]. The systematic administration of G-CSF may have contributed to the faster recovery of neutrophils, but it did not affect platelet recovery. In addition, we did not observe any primary graft failure. Similarly, Dulery et al. found that reducing the PTCY dose in the context of haploidentical transplantation resulted in faster hematopoietic engraftment [22].

PTCY has been identified as an independent risk factor for bacterial bloodstream infection during early post-transplantation follow-up [8]. In our previous study, it was found that PTCY 50 mg/kg/day was associated with a day +30 incidence of bacterial bloodstream infection of 43.2% [30]. The present results indicate that using PTCY 40 mg/kg/day can lower the risk of bacterial bloodstream infection, with a cumulative incidence within the first 100 days of 13.6%. We also observed that PTCY 50 mg/kg/day significantly prolongs patient hospitalization (median of 37 days) [30]. Based on the results of this pilot study, it appears that PTCY 40 mg/kg/day together with the addition of G-CSF may decrease hospitalization time to a median of 32 days.

The administration of G-CSF was systematically implemented from day +7 until neutrophil recovery to shorten the aplastic phase and reduce early infectious complications. G-CSF is a key therapy in hematological settings, as blocks apoptosis, stimulates cell division, and enhances granulopoiesis, thereby reducing both the duration and severity of neutropenia to prevent infections. G-CSF administration help to reduce the median of time to neutrophil recovery and indirectly bloodstream infection risk. In addition, G-CSF administration did not impact GVHD incidences or the onset of other vascular endothelial post-transplant complications, suggesting that its administration is safe in the alloHSCT setting conducted with PTCY [31,32,33]. Notice however, that these finding should be considered preliminary secondary to the reduced sample size of patients included in this study and would have to be confirmed in future investigations.

Early cardiotoxicity induced by PTCY is also a matter of concern. Between 7.4% and 19% of early cardiac events have been reported with the use of PTCY [11,12,34]. In our series, with 72.7% of patients previously exposed to anthracyclines, the incidence of early cardiac events was lower (4.5% at 2 years) than that reported by other authors [11,12,34].

Our study is limited by a small cohort size and relatively short follow-up period. Unlike other studies that combined reduced doses of PTCY with multiple immunosuppressive drugs, our research utilized only Tac alongside PTCY, achieving effective prevention of acute GVHD. We plan further analysis with a larger patient sample and extended follow-up to validate these initial findings. Before broadly implementing reduced doses of PTCY, especially in regimens that pair it solely with Tac, a pilot study is crucial. This preliminary study assessed the feasibility and refined the research design, serving as a foundational step for larger-scale applications. Pilot studies are essential as they evaluate the practicability and fine-tune methodologies for robust outcomes in future comprehensive research. It is important to analyze and share results from the initial patient group enrolled under this protocol. Based on these early observations, the protocol remains active at our institution, allowing for the enrollment of additional patients. Our objective is to conduct a follow-up analysis with a larger cohort and longer follow-up to confirm the pilot study’s preliminary results.

## 5. Conclusions

In conclusion, this pilot study shows that PTCY 40 mg/kg/day on days +3/+4 together with Tac appears to be an acceptable dose reduction in the setting of PBSC MRD and MUD alloHSCT. This strategy may reduce toxicity and improve hematopoietic and immune reconstitution while maintaining GVHD prevention.

## Figures and Tables

**Table 1:** Patient and transplant characteristics.

Patient Characteristics	Nº (%)
**Age at transplant**	
Median (range), years	53 (38–62)
**Sex**	
Male/Female	16 (73%)/6 (27%)
**Diagnosis**	
Acute myeloblastic leukemia	12 (54%)
Myeloproliferative disorders	5 (23%)
Myelodysplastic syndrome	2 (9%)
Non-Hodgkin lymphoma	2 (9%)
Acute lymphoblastic leukemia	1 (5%)
**Disease status**	
First complete remission	9 (41%)
>Second complete remission	3 (14%)
Partial remission	6 (27%)
Refractory disease/Stable disease	4 (18%)
**Disease Risk Index**	
Very high	1 (5%)
High	8 (36%)
Intermediate	6 (27%)
Low	3 (14%)
Unclassifiable	4 (18%)
**Conditioning intensity**	
RIC	11 (50%)
MAC	11 (50%)
**Detailed information on Conditioning Regimens**	
Reduced-intensity regimens	
FLAG/IDA/Bu2	2 (9%)
FLAG/IDA/Treo	2 (9%)
FLAG/Bu	1 (5%)
Flu + Bu (9.6 mg/kg)	6 (27%)
**Myeloablative regimens**	
Flu + Bu (12.8 mg/kg)	8 (36%)
Flu + TBI (12Gy)	2 (9%)
Flu/Treo	1 (5%)
**Hematopoietic cell transplantation-comorbidity index**	
≤3	8 (36%)
>3	14 (64%)
**Karnofsky performance status**	
≤80	15 (32%)
**Donor type**	
Match related donor	8 (36%)
Match unrelated donor	14 (64%)
**Donor/recipient gender mismatch**	
Female/Male	6 (27%)
Dose of CD34^+^ cells (×106/kg), median (range)	6.25 (4.96–7.18)
**Cytomegalovirus risk**	
High risk	3 (14%)
Intermediate risk	17 (77%)
Prophylaxis with letermovir	18 (82%)

FLAG/IDA/Bu, fludarabine, cytosine arabinoside, idarubicin, busulfan; FLAG/IDA/Treo, fludarabine, cytosine arabinoside, idarubicin, treosulfan; FLAG/Bu, fludarabine, cytosine arabinoside, busulfan; Flu, fludarabine; TBI, total body irradiation; RIC, reduced-intensity conditioning; MAC, myeloablative conditioning.

**Table 2:** Summary of the results of studies using reduced doses of PTCY with/without ATG in the setting of HLA-mismatched, MRD, and MUD alloHSCT.

Author	n	Type of Donor	PTCY Dose	Other Immunosuppressant Drugs	II–IV Acute GVHD *	III–IV Acute GVHD *	All-Grade Chronic GVHD **	Moderate–Severe Chronic GVHD **
Juárez et al.	22	MRD, MUD	40 mg/kg/day +3/+4	Tac	18.2%	4.5%	11.4%	6.4%
Sun et al. [25]	51	MRD, MUD	50 mg/kg/day +3	ATG + CsA + MMF	6.2%	0%	11.5%	NR
Zu et al. [26]	53	MRD MUD	20 mg/kg/day +3/+4	ATG	24.5%	NR	14.1%	NR
Solterman et al. [27]	22	1-antigen MMUD	40 mg/kg/day +3/+4	CsA + MMF/MTX	15%	NR	26%	NR
Zhang et al. [28]	29	HLA-identical donors	3 + 3 design trial DL1: 50 mg/kg/day +3/+4 DL2: 50 mg/kg/day +3 and 25 mg/kg/day +4 DL3: 25 mg/kg/day +3/+4	CsA	28.6% (for 21 patients with DL3)	0% (1 patient at day +141)	37.3%	16%
García-Cadenas et al. [29]	14	MRD MUD 1-antigen MMUD	30 mg/kg/day +3/+4	Tac	28.6%	7%	36%	14%

* Cumulative incidence at day +100. ** Cumulative incidence at 1-year; NR, Not Reported; DL, dose level.

## Data Availability

The data supporting this study’s findings are available on request from the corresponding author.
